# Recommendations for Control of East African Sleeping Sickness in Uganda

**DOI:** 10.4103/0974-777X.59250

**Published:** 2010

**Authors:** Simon Kotlyar

**Affiliations:** *Department of Emergency Medicine, Yale University School of Medicine, New Haven, CT, USA*

**Keywords:** East African sleeping sickness, insecticide-treatment, animal reservoir, monitoring and evaluation

## Abstract

East African sleeping sickness, caused by *Trypanosoma brucei rhodesiense*, is prominent in Uganda and poses a serious public health challenge in the region. This publication attempts to provide key components for designing a strategy for a nationwide initiative to provide insecticide-treatment of the animal reservoir to control *T. b. rhodesiense*. The contents of this article will focus on insecticide-based vector control strategies, monitoring and evaluation framework, and knowledge gaps required for future initiatives.

## INTRODUCTION

East African sleeping sickness, caused by *Trypanosoma brucei rhodesiense*, is prominent in Uganda and poses a serious public health challenge in the region. Epidemics have occurred during 1901 – 1915, 1940 – 1946, and 1976 – 1989.[1] More recently, the spread of sleeping sickness to areas previously thought to be free from the disease has heightened the need for effective control strategies in order to prevent future outbreaks and limit the sylvatic and human foci of transmission.[2] Sporadic outbreaks of sleeping sickness have recently been observed outside the historical geographic borders of southeast Uganda, with reports in the Soroti District being linked to introduction of the disease through cattle restocking from the endemic southern districts.[2] In addition, historical data has demonstrated a spread of sleeping sickness at a rate of 5 km per year over the past 34 years in southeastern Uganda.[1] These trends suggest a change in human-vector interaction and / or an introduction of parasites into previously unaffected regions. Recent data suggest that internal displacement, collapse of control programs, environmental modification and livestock migration are the principle factors contributing to the aforementioned trends.[1–5]

The purpose of this review is to highlight the key components for the design of a national control program for insecticide treatment of the animal reservoir to control *T. b. rhodesiense.* This report will focus on insecticide-based vector control strategies, monitoring and evaluation framework, and knowledge gaps required for future initiatives.

## BACKGROUND METRICS

*T. b. rhodesiense* Human African Trypanosomiasis (HAT) has been endemic in southern and eastern Uganda for over 100 years and is zoonotic, with a reservoir in wild animals and domestic livestock. The parasite is transmitted by Glossina tsetse flies, which are strongly zoophilic and take only a small percentage of blood meals (16 – 23%) from humans.[6] Data from previous studies in southeast Uganda reported the prevalence of *T. brucei* species in the domestic cattle population to be 5%, with 23% of these comprising the human infective form, T. b. rhosesiense.[7]

**Figure 1:**
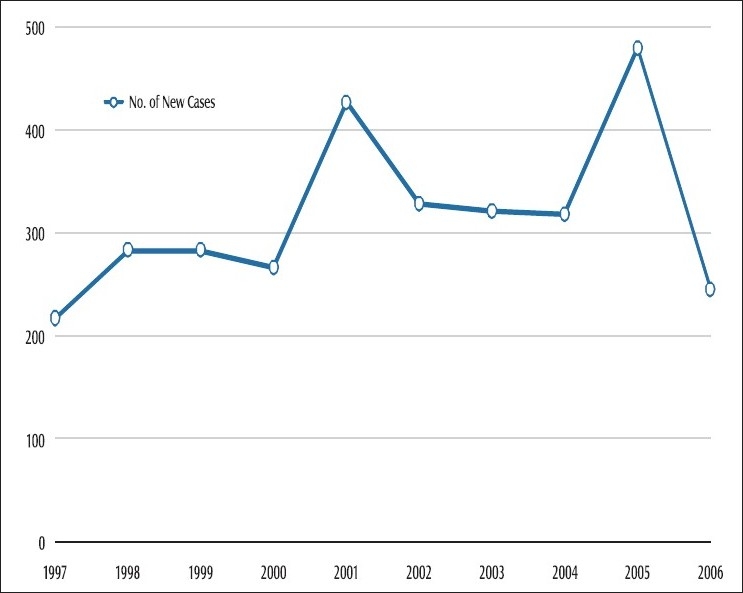
New *T. b. rhodesiense* cases reported to WHO, Uganda 1997-2006. (WHO 2007)

The National Sleeping Sickness Control Program, at the Ugandan Ministry of Health, has collected surveillance and primary data on sleeping sickness incidence in Uganda since 1999. Retrospective cross-sectional data has been collected for the period 1973 – 2003 from district summaries and record books. Reliability of the data is considered moderate to high for 1986 – 2003; however, estimates probably grossly underestimate the true disease incidence, with an estimated 12 undetected sleeping sickness deaths for every one reported.[8] Cluster-detected cases of sleeping sickness in southern Uganda for 1973 – 2003 are presented in Table 1. Recent data for new *T.b. rhodesiense* cases in Uganda reported to the World Health Organization (WHO) for 1997 – 2006 [Figure 1], show a relatively stable incidence of reported disease. A total of 14 districts were affected with a notable northward extension of cases.[9]

**Table 1:** Cluster detection of sleeping sickness. Southeastern, Uganda, 1970-2003

Interval (cluster)	Districts in most likely cluster	Cluster date	No. Observed cases	No. Expected cases	Relative risk*	*P* value	Cluster radius (km)
1970-1975	Mayuge, Bugiri, and southern Iganga	1973-1975	63	8	8.3	0.0001	41
1976-1979	Northwest shift to include northern Mayuge, lganga, Jinja, and southeastern Kamuli	1978-1979	311	23	13.5	0.0001	29
1980-1988	Wider extent, including Mayuge, Bugiri, Iganga, Jinja, and southern Kamuli	1985-1988	13,943	1,865	7.5	0.0001	45
1989-1997	As above, plus Tororo, Busia, eastern Mukono, southern Kayunga, and southern Pallisa	1989-1992	3,176	869	3.7	0.0001	74
1998-2003 (A)**	Northwestern Iganga (Luuka county) and southern Kamuli	1999-2001	331	26	12.6	0.0001	19
(B)**	Soroti	2001-2003	263	21	12.5	0.0001	22
(C)**	Tororo (Osukulu subcounty)	2001-2002	89	7	12.9	0.0001	6
(D)**	Mukono (subcounties of Buikwe, Buyikwe, Najja, Ngogwe, and Ssi)	1998	50	4	12.5	0.0001	0***

Source: Berrang-Ford, *et al*. 2006,

* Observed no. cases/expected no. cases,

** Multiple clusters were identified during 1998-2003,

*** Cluster included only 1 observation representing 5 merged subcounties.

Outbreaks of HAT in previously unaffected northern districts have been linked to cattle migration and human proximity to the animal reservoir.[2] In one case-control study of 17 villages in southeast Uganda, risk factors for HAT included: having a family member with a history of HAT (OR 16.23 CI95 2.97 – 88.74), presence of cattle around the homestead (OR 3.08 CI95 1.16 – 8.14), homestead work (OR 12.52 CI95 1.48 – 106.4), wetland buffer < 500 m (*P* < 0.05), and male sex (63%).[5]

The effects of trypanosomiasis extend beyond that of HAT, as the veterinary implications have substantial effects on the livelihood and stock of rural farmers. Animal trypanosomiasis reduces the offtake of animal protein, decreases milk production, and is estimated to cost livestock producers and consumers US $1.3 billion annually.[3,10] Veterinary control of trypanosomiasis, at the level of the individual farmer, is likely to have a duel benefit of reduced *T. b. rhodesiense* transmission in man and improved livestock livelihood.[11,12] As buy-in from farmers who already routinely treat livestock with trypanocides comes with a limited need for incentivisation, the potential for the scale-up of insecticide-based control measures in the animal reservoir is substantial.

## CONTROL STRATEGIES

Control measures for sleeping sickness have met significant sustainability challenges. The main large-scale strategies that have been used include: (1) removal of susceptible human populations from risk areas, (2) mass chemoprophylaxis of human populations, (3) active surveillance and treatment to control existing human infections. These direct human interventions have been accompanied by attempts to reduce vector populations by habitat modification and tsetse trapping.[3] Approaches based on elimination of T. brucei from the animal reservoir have been largely experimental and have shown significant promise.[13–16]

**Figure 2:**
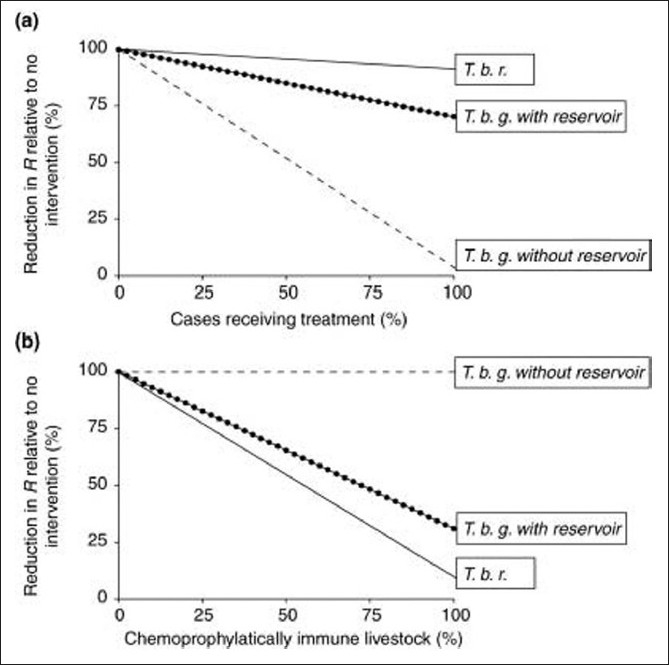
Experimental model for reduction of reproductive rate (R0) for case treatment vs. livestock chemoprophylaxis. T.b.r = *Trypanosoma brucei rhodesiensse*. T.b.g. = *Trypanosoma brucei gambiense*. Adapted from Welburn *et al*. 2001.

While it has been shown that insecticide treatment is effective in the reduction of tsetse fly populations and concomitant *T. brucei* infection in animals, the effects on *T. b. rhodesiense* infection in humans has not been clearly demonstrated.[13–16] Similar approaches for control of malaria in human population through insecticide treatment of cattle have proven effective in reducing Plasmodium infection in areas with zoophilic Anopheles species.[17] In addition, mathematical models have demonstrated a limited reduction in the basic reproductive rate (R0) for *T. b. rhodesiense* with case treatment, and a near linear reduction of R0 with chemoprophylaxis of the animal reservoir, suggesting that the optimal approach to control east African sleeping sickness may lie in curtailing disease in the animal reservoir [Figure 2].[4]

Synthetic pyrethroids have been used to control a wide range of ectoparasites in cattle since the 1970s.[18] Of those synthetic pyrethroids screened, deltamethrin appears to be the most potent, photostable, and persistent, with safety and efficacy at very low dosages.[14] Its effects on controlling tsetse flies, ticks, and mosquitoes is well-documented and cattle dipping with deltamethrin has been established as an acceptable form of vector control.[14,17,19,20] A multitude of variables affect the efficacy of deltamethrin application in cattle to control the tsetse fly vector in areas of high fly burden. Nonetheless, deltamethrin treatment has been seen to reduce tsetse burdens by 70 – 100% within three to six months in a number of settings.[14,21–23] In addition, it has been seen that deltamethrin reduces bovine trypanosomiasis by 40 – 98%.[21,24] Cost effectiveness can be significantly increased by restricted application of the insecticide to the belly and legs of cattle without a notable decrement in efficacy.[25,26]

The methods and protocols used for deltamethrin application to cattle are variable and depend on the manufacturers' specifications, concentration, temperature, and seasonality (dry vs. rainy season). The potential added benefits of application include reduction of tick-borne and mosquito-borne diseases, improved health of livestock, and increased yield from husbandry.

## KNOWLEDGE GAPS

Although the effects of insecticide-based vector control strategies in cattle are known to decrease tsetse fly burden and bovine trypanosomiasis, the effects on the human form of the disease have not been elucidated.[14,21–24] It is assumed that reducing the vector burden and decreasing the incidence of disease in the animal reservoir will translate into beneficial effects in reducing the incidence of disease in man. However, whether this will decrease the force of infection sufficiently or not remains to be determined and depends on the degree of coverage in the reservoir. Questions remain regarding the role of human-to-human transmission, the extent to which other animal reservoirs play a role, and the emerging potential for insecticide resistance. Deltamethrin and other insecticides have been shown to reduce the incidence of malaria in man when applied to cattle in zoophilic foci and a similar effect is postulated for HAT control in East Africa.[17]

The following research questions regarding Glossina control in Uganda can assist in future planning:

1. Does insecticide-based vector control in cattle reduce the incidence of *T. b. rhodesiense* in the human population?
2. What is the cost effectiveness of mass insecticide treatment?
3. Is mass treatment of livestock acceptable to local herdsmen and farmers in Uganda?
4. What is the prevalence of *T. b. rhodesiense* in other domestic and sylvatic animal reservoirs?
5. Should similar approaches with insecticide treatment be extended to other domestic livestock?
6. What is the evidence for Glossina species resistance to common synthetic pyrethroids?

Questions 1 – 3 should be elucidated prior to embarking on a nationwide control program. The remaining questions may fall within the context of data collection and evaluation during the implementation phase of a program. Given the extensive zoonotic distribution of *T. brucei rhodesiense*, eradication of the parasite is highly unlikely. Future research should focus on effective methods of vector control, cost-effective disease surveillance, and early case detection and treatment.

## RECOMMENDATIONS

Long-term recommendations for an insecticide based vector control program rely on successful implementation of pilot-site programs and will depend on local factors and variables identified during pilot program implementation. Larger rollout and effective scale-up of existing ground level programming should proceed when efficacy and feasibility studies are available. The recommendations that follow are for such an initial pilot-program, which should form the framework for a ground-up approach to successful countrywide implementation.

**Figure 3:**
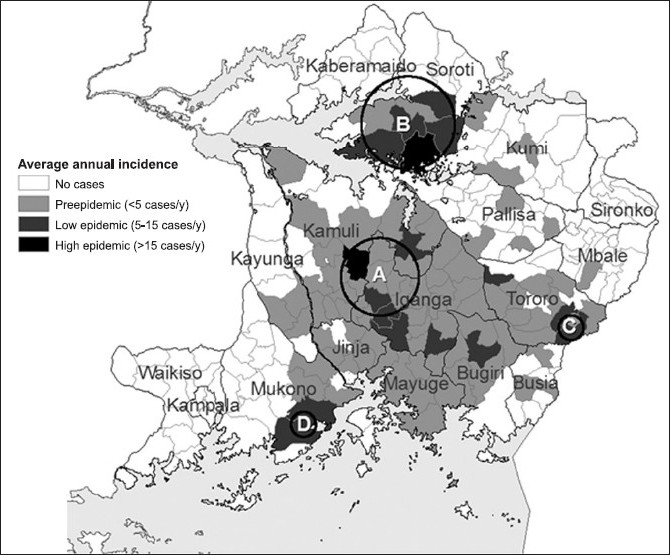
Average annual incidence of sleeping sickness by sub-county, 1998-2003. Berrang-Ford et. al. 2006

Initial pilot programs should be targeted to epidemic foci of disease. Spatial analysis of disease incidence, from 2003, has identified five districts with epidemic pockets of *T. b. rhodesienses* [Figure 3]. Initial pilot programs for insecticide treatment of cattle should be targeted at these five districts (Mukono, Iganga, Tororo, Kamuli, and Soroti). District health teams responsible for program implementation should be tasked with coordination of county and sub-county level dispensing, education campaigns, data collection, and reporting.

A regional task force should be convened consisting of county level public representatives, public health experts, veterinarians, medical providers, non-governmental partners, and Ugandan Ministry of Health (MoH) leadership.

The task force should seek to establish a detailed methodology for such pilot-level programs, consisting of:

1. Detailed methodology and analysis plans for components outlined in the Monitoring and Evaluation framework
2. Timeline and methods for sub-county and village level implementation
3. Requisite materials and supplies list
4. Deltamethrin formulary and recommended application methodology
5. Estimate of district level budgets

On completion of initial pilot-phase programs, an analysis of efficacy and cost-effectiveness should be determined. Based on surveillance data from other districts, information obtained from pilot programs and research inputs, as outlined in the knowledge gaps section, a long-term decision regarding feasibility and strategy can be made.

## MONITORING AND EVALUATION FRAMEWORK

The effective implementation and long-term feasibility of an insecticide-based vector control program relies heavily on the ability to successfully implement a reliable and consistent monitoring and evaluation framework. Detailed plans and methods should be constructed for the following metrics:

1. Epidemiological monitoring:
  - Number of reported sleeping sickness cases in pilot areas (incidence)
  Successful monitoring of sleeping sickness cases requires effective diagnostic capacity and reliable case detection with microscopic identification of trypanosomes in blood, lymph, and cerebrospinal fluid. As there is no seroscreening test available for *T. b. rhodesiense*, sensitivity analysis and quality control measures are limited.[27]
2. Entomological monitoring:
  - Tsetse fly population density
  Standardized box-trap methods for uniform monitoring of tsetse fly population should be applied at regular intervals. Correlation to seasonal and livestock migration patterns should be uniformly applied.
3. Veterinary monitoring:
  - Prevalence of bovine trypanosomiasis
  - Weight changes and milk yield in cattle
  A random cohort of cattle should be monitored for serologic evidence of bovine trypanosomiasis in the pre- and post-treatment phases. Regular monitoring of weight and milk yield should also be sampled during the pilot period.
4. Disease surveillance:
  - Countrywide sleeping sickness incidence
  - Investigation and control of regional outbreaks
  Disease surveillance carried out by the National Sleeping Sickness Control Program, Ugandan Ministry of Health, should be integrated with the analytical framework for the vector-control program. Common reporting structures and databases should be utilized.
5. Population acceptance and attitudes:
  - Farmers perception of productivity impact
  - Willingness to treat animals with insecticide
  - Benefit to community
  Questionnaires should be developed based on local input following focus groups and identification of barriers and limitations. Metrics specific to knowledge, attitude, and practice should be developed to address the farmers' perception of productivity impact, willingness to treat, and benefits to community.
6. Environmental impact:
  - Concentration of deltamethrin in regional water supplies
  Given the nature of the proposed intervention, the potential for introduction of large quantities of synthetic pyrethroids into soil and water supplies exists. Water quality guidelines for Deltamethrin, set a safety limit at 0.0004 μg per L.[28,29] Based on expert consensus and recommendations, a regular evaluation of water supplies should be carried out, to assess the potential environmental impact on freshwater fish and agricultural symbiotic species.

## CONCLUSION

The effective control of East African sleeping sickness is challenging given the nature of vector-host-reservoir interactions. Insecticide treatment of the domestic animal reservoir may provide for a reduction in disease incidence in man and may halt the extension of the disease into previously unaffected regions. Deltamethrin application to cattle reduces tsetse fly burden and bovine trypanosomiasis. Further research is necessary to determine if such interventions have the potential to reduce HAT in man and if such an approach is cost-effective. Pilot programs should be launched in the epidemic foci of *T. b. rhodesiense* transmission. Core metrics should be collected to estimate the impact and feasibility of a nationwide, vector-control strategy, to reduce the incidence of East African sleeping sickness, in Uganda.
